# Zimmermann-Laband syndrome and infantile systemic hyalinosis: an enigma with two separate terms with overlapping features: a case report

**DOI:** 10.1186/s12887-023-04344-z

**Published:** 2023-10-13

**Authors:** Fatemeh Owlia, Alireza Navabazam, Mohammad-Hasan akhavan-karbasi, Mohammad Moein Derakhshan Barjoei

**Affiliations:** 1 Department of Oral and Maxillofacial Medicine, School of Dentistry, Yazd Shahid Sadoughi University of Medical Sciences and Health Services, Yazd, Iran; 2 Department of Oral and Maxillofacial Surgery, School of Dentistry, Yazd Shahid Sadoughi University of Medical Sciences and Health Services, Yazd, Iran; 3 Dentistry student, Student Research Committee, Shahid Sadoughi University of Medical Sciences, Yazd, Iran; 4 USERN Office, Shahid Sadoughi University of Medical Sciences, Yazd, Iran

**Keywords:** Case report, Gingival enlargement, Infantile systemic hyalinosis, Papulonodular lesions, Zimmermann-Laband Syndrome

## Abstract

**Background:**

Zimmermann-Laband Syndrome (ZLS) and infantile systemic hyalinosis (ISH) are rare genetic disorders. They are characterized by various spectrum manifestations. In spite of other case reports, this case with features of both syndromes was reported by oral medicine specialists and oral and maxillofacial surgeons.

**Case presentation:**

In this study, we reported an 18-months old female patient with gingival overgrowth. This phenomenon completely embedded all the erupted teeth. In this case, the presence of multiple papulonodular cutaneous lesions is a newly observed aspect that has rarely been reported in the existing literature. Gingival overgrowth was excised under general anesthesia. At six months of follow-up after surgery, mastication and breathing problems were improved. Aesthetic aspects were ameliorated in terms of gingival appearance.

**Conclusions:**

To date, due to the ambiguous presentations, both syndromes remain an enigma for specialists. A timely diagnosis could be crucial for prognosis and preventing severe further surcharge. Dentists could play an important role in the diagnosis of rare disorders.

## Background

Infantile systemic hyalinosis (ISH) and juvenile hyaline fibromatosis (JHF) are two variants of Hyaline fibromatosis syndrome (HFS). JHF is the mild form, and ISH is the severe form [1]. Non-cancerous papulonodular lesions are the hallmark of the diagnosis of ISH. The appearance of lesions in areas of mechanical stress may suggest that microtrauma repair mechanisms are involved in developing these lesions. The treatment for ISH aims to improve the patient's quality of life through palliative care [2].

Infantile systemic hyalinosis (ISH) is a genetic condition that is inherited in an autosomal recessive manner. It is typically identified by the appearance of skin lesions during infancy, along with other associated symptoms. Progressive deposition of amorphous hyaline material in different tissues of vital organs could lead to various presentations. Infantile Systemic Hyalinosis (ISH) typically manifests at birth or during the initial months of life. This condition is often diagnosed by reduced spontaneous movements due to progressive joint contractures [3].

The most important clinical manifestations of ISH are progressive skin thickening, short stature, gingival hypertrophy, hyper-pigmented patches on bony prominences, and fleshy nodules (particularly in the perianal region), and the presence of Osteopenia may lead to increased susceptibility to bone fractures. Children affected by deposition disorders are at risk of frequent infections and/or intractable diarrhea, which is due to protein-losing enteropathy. Unfortunately, these children often pass away in infancy due to organ failure [1].

Zimmermann-Laband Syndrome (ZLS) is a sporadic craniofacial malformation syndrome characterized by specific oral features of diffuse gingival fibromatosis in early childhood [4, 5]. Its prevalence is reported to be less than 1/1000000 of the population [6]. The syndrome is characterized by various symptoms, including gingival fibromatosis, seizure, dysplastic or absent fingernails, hypertrichosis, hirsutism and abnormalities in nose and/or ears anatomy [7]. Other reported symptoms related to the syndrome in the literature include colpocephaly, hemivertebra, polydactyly, segmental hyperpigmentation, and hemi facial hyperplasia, which expand the phenotypic spectrum of the disease [8, 9]. Reports indicate that the cardiovascular, gastrointestinal, and nervous systems are also affected. Many patients experience cosmetic and functional issues due to hereditary gingival fibromatosis [10].

Researchers mentioned facial and extremities disorders involved ear thickness, cartilage softness, nose prominence, gingival overgrowth, nail aplasia or hypoplasia, and type B brachydactyly. Other studies reported hirsutism, mental disability, and visceral organ involvement. A wide range of clinical manifestations were reported. Some cases were diagnosed during childhood, while others were already adults [9, 11, 12]. Mental retardation was present in some cases, while others had an average IQ score. The primary differential diagnosis for ZLS includes variants of Hyaline Fibromatosis Syndrome, syndromes with Gingival Fibromatosis, and other similar disorders. Gingival hypoplasia of the distal phalanges, scoliosis, abnormal fingers, hepatomegaly/splenomegaly,

fibromatosis (GF) is a rare condition that may be linked to specific syndromes. It can cause excessive growth of gum tissue, which may partially or entirely cover teeth. Clinicians can benefit from understanding the differential diagnosis of gingival fibromatosis, as it can aid in distinguishing ZLS through a more straightforward approach [13, 14]. Genetic evaluation in ZLS was also applied for research goals [7, 15]. Typically, ZLS is inherited through an autosomal dominant trait, though there have also been reports of autosomal recessive inheritance [16].

In severe situations, it may be necessary to surgically remove the hyperplastic fibrous tissue to uncover teeth that are embedded [17]. While the syndrome is not fatal, the prognosis is based on the severity of the manifestations [2].

The present study aimed to report an 18-month-old female patient with overlapping features of ZLS and ISH. A literature review was conducted to gather the latest information about these syndromes and provide insights into their clinical presentations. To obtain the related articles, international databases of PubMed and Scopus were searched with defined structural MeSH and non-MeSH keywords infantile systemic hyalinosis, Hyaline fibromatosis syndrome, papulonodular lesions, and Zimmermann-Laband Syndrome. This particular case has been reported by specialists in oral medicine and oral and maxillofacial surgery, despite previous cases reported by other medical specialties. The case highlights the significance of gingival fibromatosis as a diagnostic milestone and can assist clinicians in accurately diagnosing and distinguishing similar differential diagnoses.

## Case presentation

**Fig. 1:**
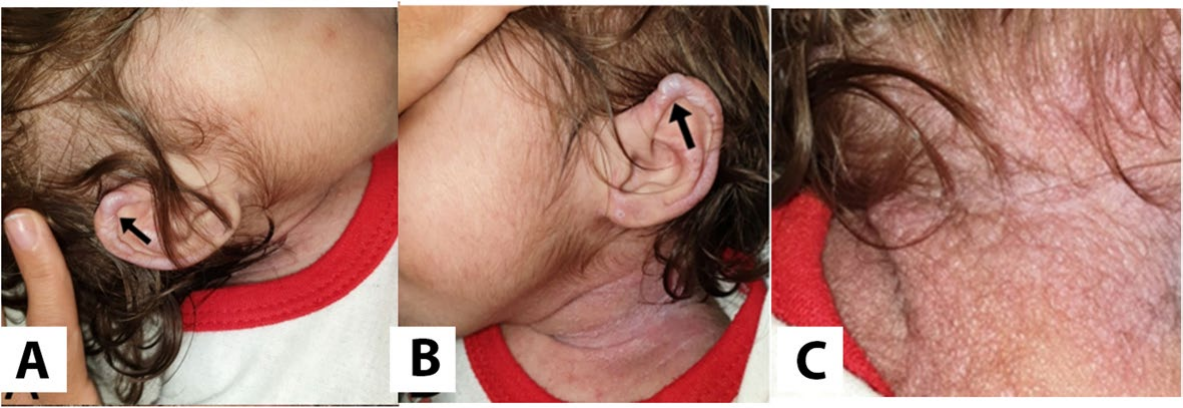
Head and neck features. **A** and **B**) Diffused papulo-nodular lesions on ear helixes. **C** Multiple papulo-nodular lesions on neck

An 18-month-old female patient was referred to an oral medicine specialist due to gingival enlargement. The chief complaint was an excessive gingival overgrowth of the anterior region of the mouth, which had covered all deciduous teeth eight months ago (Fig. 1). The patient was born to a 25-year-old woman and her healthy 38-year-old husband, who were in a consanguineous marriage. The patient was their only child.

Her parents appeared healthy. The mother experienced pregnancy without any complications or noteworthy events. Her birth history indicates that she was born at full term (39 weeks) through expected spontaneous vaginal delivery.

According to the parents’ information, one of their siblings died at the age of 6 months. The cause of his death was severe diarrhea with no definite diagnosis. They stated that he had pigmented patches on his knees, similar to the patient. The dead child seemed to have the same disorder as the reported patient.

Her growth and development were regularly evaluated and compared to typical growth charts throughout the first four months of the patient's life. However, at six months, the child began to display skin-related symptoms and signs of flexion deformities. As a result, the patient was admitted for further investigation regarding muscle hypotonia, recurring diarrhea, and diagnostic workup. While laboratory tests were within the normal range, mild leukocytosis, anemia, and C-reactive protein(CRP) = 3 + were noted. Additionally, the patient's medical history highlighted recurrent chest infections.

**Fig. 2:**
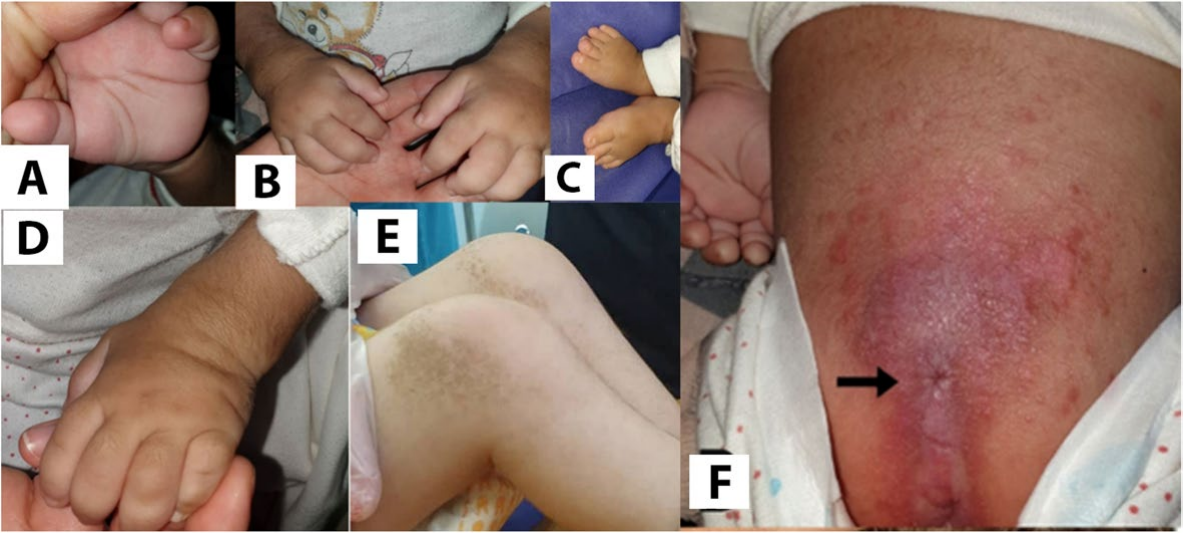
**A**-**D**) Extremities feature. **E** Contractures and symmetrical skin patches around knees, **F**) The multiple papula-nodular lesions in the napkin area

Clinical examination revealed short stature, hypoplasia of the distal phalanges, scoliosis, and hepatosplenomegaly. On cutaneous examination, hypertrichosis was found. Diffused violaceous papula-nodular lesions were observed on her ear helixes, neck, and napkin area, and Contractures and flexion deformity presented at both knee joints (Fig. 2).

At 20 months, the child's developmental monitoring revealed delayed growth. She could not sit, stand, walk, or crawl on all fours and could only speak a few words. However, her intelligence quotient (IQ) test indicated an average score. Hearing loss was ruled out based on the results of Transient-evoked otoacoustic emission (TEOAE) and OSM tests. More details are noted in Table 1.

**Table 1:** Clinical manifestations

Signs of the syndrome	Body skin	Mental retardation	Gastrointestinal system	Head and face	Oral	Neck	Extremities and skeletal system
Type of involvements and locations	Multiple popular (on the neck, back, and lumber) Populonodular (body skin)	No	Diarrhea but no specific disease	Multiple rashes (around the nose) Popular firm lesions resembling pearl-shape, keratolytic psoriasis (ear helix) Wide nasal ridge	lower lip protrusion alveolar ridge swelling open mouth Embedded erupted teeth under the gingiva	Plaque–like lesions with non-smooth surface and erythematous base (mostly on posterior site)	Three main palmar lines (absence of other palmar lines) Lumber deformity halluces hypotonic myopathy joint stiffness dysarthria

**Fig. 3:**
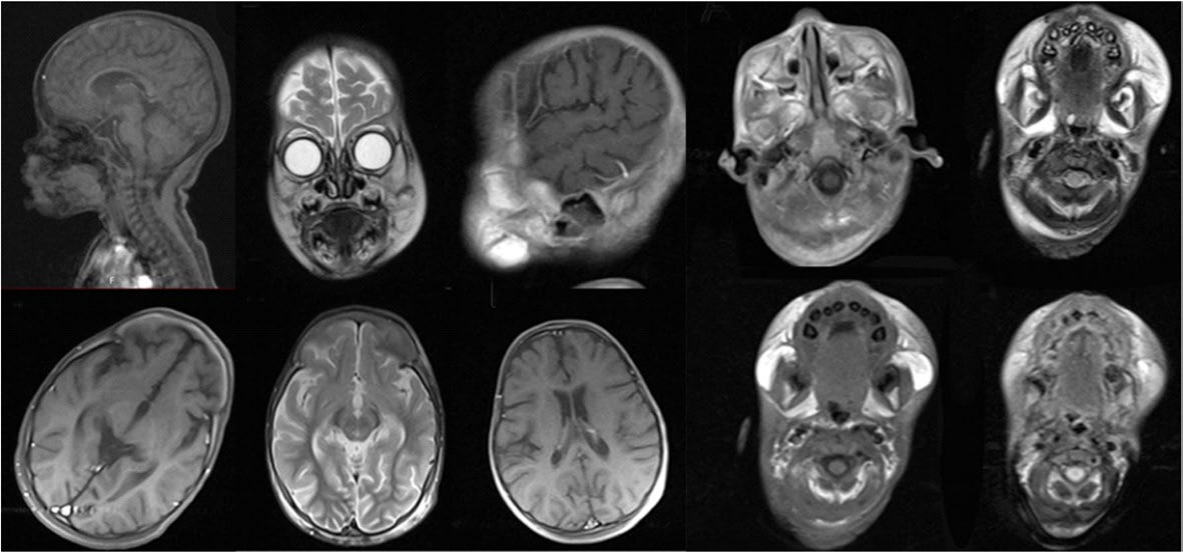
Head and neck MRI findings. No specific abnormal findings were seen in MRI. The condition of cerebral ventricles and other components is normal

Head and neck examination revealed facial abnormalities composed of saddle nose and bilateral nodules around the nasal alae and over the ears. Swollen lips, hirsutism, hypertelorism, and coarse hair are shown in Figs. 1 and 3.

Intraoral examination revealed maxillofacial deformity, an extensive pale pink firm enlargement of gingiva involving the maxillary and the mandibular arches, alveolar ridge swelling, non-plaque induced gingival diseases and conditions, and anterior open bite. All erupted anterior teeth were covered entirely with gingiva fibromatosis. This growth had a monotonous base with a lobular surface. The palate was free of any lesions.

Upon reviewing her system, it was discovered that she had a limited range of motion in both her upper and lower limbs and muscle weakness. Ultrasonography confirmed the presence of hepatosplenomegaly, and she also had two symmetrical skin patches around her knees. However, her cardiac evaluation was found to be regular. Additionally, a brain MRI with and without contrast was reported as normal (Fig. 3).

**Fig. 4:**
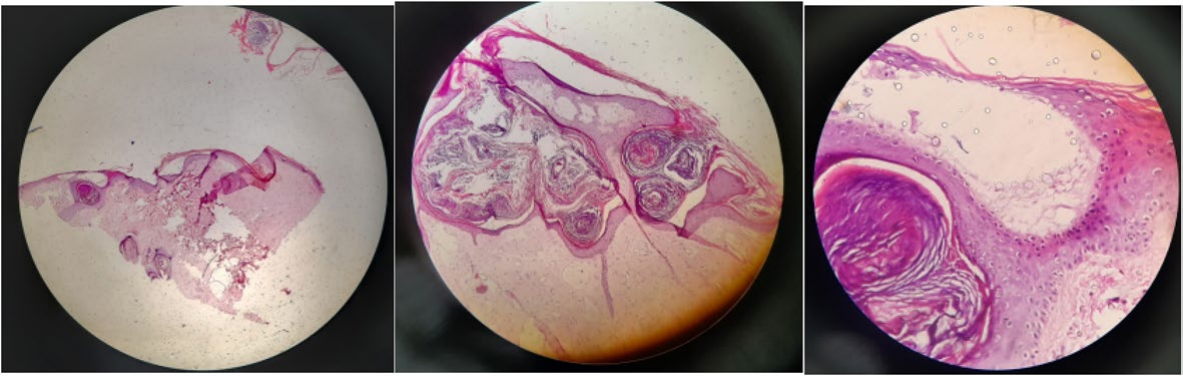
Histopathologic picture from × 4, × 10, and, × 40 magnification. In this view the hyalinization of epithelium, fusiform and round cells was founded

**Fig. 5:**
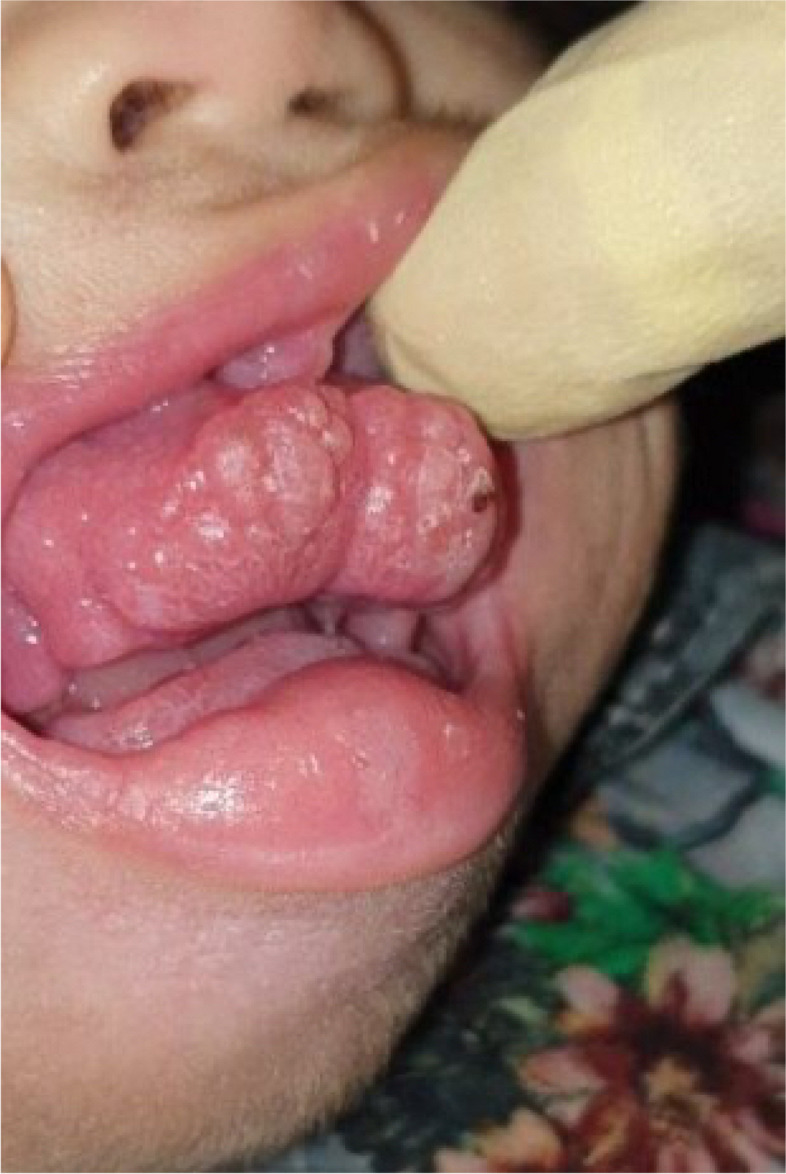
Gingival fibromatosis view of the patient

Usually, clinical documents are enough to diagnose ZLS. Meanwhile, diagnosing gingival fibromatosis is typically done through a plain oral biopsy and confirmed after microscopic evaluation. An incisional biopsy of the lower lip and gingiva was performed in this case. The histological findings showed hyperplastic squamous epithelium overlying a collagenized fibrous stroma with sparse lymphocytic infiltration, which led to the diagnosis of gingival fibromatosis (Figs 4 and 5).

About one year after the biopsy, in periodic follow-ups, it was discovered that her head and neck papules had worsened, particularly on the back of her neck. Her body rigidity had also deteriorated to the point where she could not sit in a dental chair or use her hands to eat at just 2.5 years old. However, there were no signs of any mental issues, and she could speak like an average 2-year-old child.

Eight months after oral surgery, there was a relapse in lip prominence. Despite regular teeth eruption, poor oral hygiene was observed due to difficulty accessing the mouth. These new presentations appear to be linked to managing systemic conditions.

## Discussion

ZLS has overlapping manifestations with some disorders, including FHEIG syndrome. This syndrome is known for its facial dysmorphism, hypertrichosis, epilepsy, intellectual disability/developmental delay, and gingival overgrowth. However, after examining the case, it was determined that the diagnostic criteria for FHEIG syndrome did not apply, and it was ruled out as a possibility.

Dermatologists suggested ISH as the first diagnosis based on cutaneous lesions on the peri-nasal and auricle [18]. After examining the patient, the dermatologist confirmed the diagnosis of hyalinized fibrous material in nodules. This syndrome is identified by amorphous hyaline material deposited in various solid organs. Due to the severity of the disorder, it can affect multiple organs such as the gastrointestinal tract, heart, adrenals, spleen, thyroid, and adrenal glands, which can lead to multi-organ failure and potentially result in death [19].

The patient was recommended to undergo a genetic evaluation based on the results. The genetic counselor suggested the possibility of "Snijders Blok-Campeau syndrome" due to a mutation found in Exon 1 of the CHD3 gene in location NM-001005271. This syndrome exhibits neurological symptoms and affects the orofacial structures and musculoskeletal system, not the skin. Other symptoms include hypotonia, hearing loss, widely spaced eyes, midface hypoplasia, low-set ears, broad nasal base, joint laxity, and broad forehead [20]. However, we found that the patient's symptoms did not match those of this disorder.

Despite familial aggregation, different inheritance patterns have been registered. Based on current knowledge, the most confirmed ZLS is inherited in an autosomal dominant inheritance, usually resulting from a de novo mutation [21, 22]. conversely, ISH is typically inherited in an autosomal recessive manner. Nevertheless, the phenotype of another candidate gene of KDSR has been ruled out [3].

This particular condition shares similarities with ISH regarding age, skin manifestations, and orofacial expressions, particularly regarding the prominence of the lower lip. It is typically caused by the deposition of amorphous hyaline material [2]. However, our case did not exhibit the typical hyperpigmentation seen in ISH, which is usually concentrated around the metacarpophalangeal (MCP), proximal interphalangeal (PIP) joints, and the malleoli. Instead, our patient had hyperpigmentation on both knees. A comparison of three probable diagnoses and the presented case is provided in Table 2.

**Table 2:** Comparison of the case and three probable diagnoses

Syndromes			Clinical Manifestations		
	Neurology	Orofacial	Musculoskeletal system	Skin	organ involvement
Snijders Blok-Campeau syndrome [20, 23–27]	Hypotonia Widened CSF spaces (MRI) Neonatal feeding problems Microcephaly Delayed myelination Prominent extra-axial space Seizures Intellectual disability Macrocephaly Impaired speech (Delay) and language Hearing Loss Cerebral visual impairment	Widely spaced eyes Frontal bossing Full Cheeks Peri-orbital fullness Epicanthal folds Narrow palpebral fissures Deep-set eyes More prominent supra-orbital ridge Mid-face hypoplasia Low-set ears (posteriorly rotated or simple with thick helices) Broad nasal base Prominent nose Bifid nasal tip Broad nasal tip Pointy chin Sparse eyebrows Hypermetropia Strabismus Astigmatism Myopia Absent teeth Thin upper lip	Joint laxity (generalized and/or local) High forehead Broad forehead Leg deformity Prominent forehead		Males’ Genital abnormalities Patent ductus arteriosus Ventricular septal defect Atrial septal defect Hernia (inguinal, umbilical, hiatal)
Zimmermann-Laband syndrome [9, 11, 16, 28–30]	Hemi-hyperplasia Seizures Learning disability Facial palsy	Atypical retinitis pigmentosa Thick eyebrows hypertelorism Large fleshy nose Bulbous soft nose Thick floppy ears No eruption of primary teeth Hearing loss Bilateral developmental cataract posteriorly rotated ears Pit on ear helix Wide nasal bridge Mildly hypoplastic nostrils Malformed external ear lobes Massive gingival overgrowth Short philtrum Thin upper lip Gingival fibromatosis Macroglossia Supernumerary teeth Wide fissures of tounge Deep fissures of tounge Unable to close mouth Anterior end to-end bite Symmetric soft tissue swelling of the palate	Polydactyly vertebral defects Hypoplasia / aplastic of distal phalanges Hyperextensibility of joints Aplastic/hypoplastic nails Hypoplasia of the distal phalanges Joint hypermobility Short stubby fingers Absence of nails Myoclonus	Hyperpigmentation Mild hirsutism	Hepatosplenomegaly
Infantile systemic hyalinosis [31–34]	Progressive ataxia Spasticity Neurodevelopmental regression Cerebellar atrophy	Gingival hypertrophy Labial Hypertrophy Buccal hypertrophy Inability to open mouth	Synovial thickness in joint Hypertonia Joint contractures Knee pain Limited range of motion in extremities and weakness Short limbs and neck Torticollis Osteoporosis Stunted growth Osteolytic bone lesions	Papulonodular skin lesions Papuloerythematous rash Large subcutaneous tumors Hyperpigmentation Generalized stiff skin Edematous skin	Protein-losing enteropathy Diarrhea Rectal prolapse lymphangiectasia Recurrent infections Thyroid dysfunction
**Our case**	No	Multiple rashes (around the nose) Popular firm lesions resembling pearl-shape, keratolytic psoriasis (ear helix) Wide nasal ridge Lower lip protrusion Alveolar ridge swelling Open mouth Embedded erupted teeth under the gingiva Plaque–like lesions with a non-smooth surface and erythematous base (mainly on the posterior site	Three main palmar lines (absence of other palmar lines) Lumber deformity Halluces Hypotonic Myopathy Joint stiffness Dysarthria	Multiple popular (on the neck, back, and lumber) Populonodular (body skin)	Diarrhea but no specific disease

She had a gingivectomy procedure at age two to treat his gingival fibromatosis. An oral and maxillofacial surgeon carried out the surgery while she was under general anesthesia. However, six months after the surgery, the condition recurred. Despite this, she did not experience any significant issues with eating or breathing.

In other words, some diverse clinical presentations appear to be a multitude of diseases manifesting as asymptomatic carriers among the siblings and relatives of rare cases. Interestingly, the genetic carriers (with the late manifestation of the disorders) are more frequent than the subjects with the severe type of ISH and ZLS.

As far as we know, our patient is only the second case of ZLS-related symptoms diagnosed in children under the age of two. Although other medical professionals have reported similar cases, oral medicine specialists and oral and maxillofacial surgeons diagnosed this particular case based on the enlargement of the patient's gingival fibromatosis. Molecular genetic testing is currently only available for research purposes. Currently, ISH and ZLS diagnoses are based solely on clinical observations [2, 7]. Pediatricians and clinicians have underestimated the frequency of hereditary conditions like ISH or ZLS in children due to the diverse and confusing symptoms they present. These conditions progress slowly but can have devastating outcomes.

Tests for genetic syndromes that fall into the intermediary group can often provide inaccurate results, which can be frustrating for families already dealing with the long-term effects of slowly progressing genetic abnormalities that are not fully understood.

## Conclusion

In this case, the patient's symptoms fell somewhere between ISH and ZLS syndromes. However, genetic analysis results did not fully correspond to either of these conditions. It's worth noting that ZLS syndrome is still not fully understood due to its ambiguous symptoms. Nevertheless, an accurate and timely diagnosis is crucial for effective treatment and to prevent any further complications. It's worth noting that dentists can also play a significant role in diagnosing rare conditions like ZLS.

## Data Availability

The datasets are available on reasonable request to the corresponding author.

## Abbreviations

ZLS: Zimmermann-Laband Syndrome
ISH: Infantile Systemic Hyalinosis
GF: Gingival Fibromatosis
TEOAE: Transient-Evoked Otoacoustic Emission
MCP: Metacarpophalangeal
PIP: Proximal Inter Phalangeal
